# An ultrasound imaging system exploiting transducers and multiplexers on a flexible substrate together with a log-delta CMOS ADC

**DOI:** 10.1038/s41528-025-00478-5

**Published:** 2025-10-13

**Authors:** Martijn Timmermans, Kyle van Oosterhout, Marco Fattori, Paul van Neer, Pieter Harpe, Eugenio Cantatore

**Affiliations:** 1 Eindhoven University of Technology, Eindhoven, the Netherlands; 2 Acoustics & Underwater Warfare, TNO, The Hague, The Netherlands

**Keywords:** Electrical and electronic engineering, Energy efficiency, Imaging

## Abstract

Ultrasound (US) imaging is a fundamental tool in healthcare for the diagnosis of diverse conditions. Wearable, flexible ultrasound patches could expand the scope of US imaging to continuous, at-home monitoring without professional intervention, but require scaling to large numbers of transducer elements. This poses challenges in interconnect density, power consumption, and data bandwidth. To improve interconnect density, we present the first integration of flexible ultrasound transducers with flexible a-IGZO thin-film transistor (TFT) multiplexing electronics. In the Si CMOS readout chip, a new circuit technique cuts front-end power, while a log-delta ADC compresses data efficiently. Our system achieves an 8× reduction in required front-end circuitry and a 42% decrease in front-end power. The data needed to describe the ultrasound image are reduced five-fold, decreasing data transmission power by the same factor. These advances bring the vision of wearable high-density, large-area ultrasound imaging patches for monitoring one step closer.

## Introduction

The globally aging population has led to an increasing demand for hospital care, straining resources and personnel. Continuous monitoring and timely diagnosis have been shown to reduce healthcare costs and improve outcomes for pregnancy and conditions such as heart failure^1–3^. Ultrasound (US) imaging is a particularly valuable modality for this purpose, due to its widespread use for the diagnosis of various disorders of the heart and other internal organs. Advances in miniaturization have made the implementation of portable ultrasound systems possible^4–9^, and show great potential to enable the application of ultrasound technology to monitoring disease progression and treatment. However, a major limitation of current portable ultrasound probes is that they require trained sonographers to operate effectively. The process involves manual scanning, where the probe must be moved back and forth at different orientations to capture a comprehensive image. This complexity limits the practical use of portable ultrasound for untrained individuals and restricts its use to clinical and ambulatory settings only.

A revolutionary long-term vision in US technology is to enable high-quality ultrasound-based monitoring without the need for trained personnel. Ultrasound monitoring would bring important advantages in terms of timely detection of patient deterioration or critical conditions, improved patient selection for further investigations and/or interventions, earlier hospital discharges and, overall, a lower burden on the healthcare system. In our opinion, this vision can be realized by creating wearable low-cost ultrasound patches that cover sufficiently large areas (e.g. tens of cm^2^) to image organs from different view angles without human intervention or repositioning. Furthermore, the thin and flexible/conformable form factor of these patches would allow them to adapt closely to the body’s contours and maintain consistent skin contact, e.g. using adhesive dry gels^10^. Combined with AI-driven scanning that can autonomously select optimal angles, imaging sequences, and subsets of transducers to optimize power consumption and image quality, these wearable patches would be capable of continuously monitoring patients over extended periods, a significant step forward in enhancing patient care and streamlining the healthcare workflow.

Much effort is devoted in the research community to enable wearable US patches^10–20^. Many different techniques have been proposed to build US transducers on a flexible support^10,11,13–19^. However, current US patches are often read out using bulky and expensive electronic systems^21^. Recent efforts such as the Ultrasound-on-Patch (USoP) system developed at UC San Diego^6^ and the TinyProbe platform from ETH Zurich^7^ have demonstrated significant progress in miniaturized and wearable ultrasound. These systems integrate the electronics using only rigid silicon, achieving high compactness. However, in our view, their scalability to large areas with very large transducer counts in the order of 10⁵ elements is limited due to the interconnection complexity and silicon area needed. Our approach addresses this limitation by integrating flexible a-IGZO TFT-based multiplexers with flexible ultrasound transducers, and allowing the electronics to scale with the patch area while minimizing interconnects and preserving flexibility.

Achieving integration of US transducers and electronics into a cost-effective, wearable patch presents several grand challenges:

1. Connection between US transducers and readout electronics. The need for a thin, flexible and possibly conformable US patch, together with the large area (tens of cm^2^) needed to image from different perspectives organs like the heart and the uterus, translates in an extremely large number of transducer elements (~1.5 × 10^5^ on 50 cm^2^ for the 180 µm pitch used in this work) and complicates the connection between the US transducers and the silicon chips. Solutions based on monolithic integration using silicon chips^22–24^ are not flexible and are difficult to realize on a large area from both a technical and an economical perspective.
2. High front-end power consumption. Even enabling only small subsets of transducer elements depending on the chosen view, the very large number of analog front-ends needed requires significant energy, impacting battery life.
3. Data communication demands. Ultrasound imaging generates a very large amount of data (e.g. 48 Tb/s for 1.5 × 10^5^ transducer elements digitalized by 10b ADCs at the 32MS/s sampling speed used in this work). This places significant demands on the data transmitter, further increasing the energy and battery capacity needed.

Thin-Film Transistors (TFTs) represent a promising solution to address the first challenge. TFT technologies, extensively developed for display applications, offer a cost-effective method for creating transistors on a large area, even on flexible substrates^25–31^. TFTs can be used as multiplexers between US transducers and silicon chips, allowing for a great reduction of the number of interconnections, without incurring prohibitive costs. Furthermore, US transducers can be built on a flexible substrate^32^ using techniques that should be compatible with TFT technology. This integration would enable a fully flexible US patch that supports the required 10^5^ or more interconnections to the transducers, while requiring far fewer interconnects to the silicon electronics and thus significantly less silicon area, reducing cost and complexity.

**Fig. 1:**
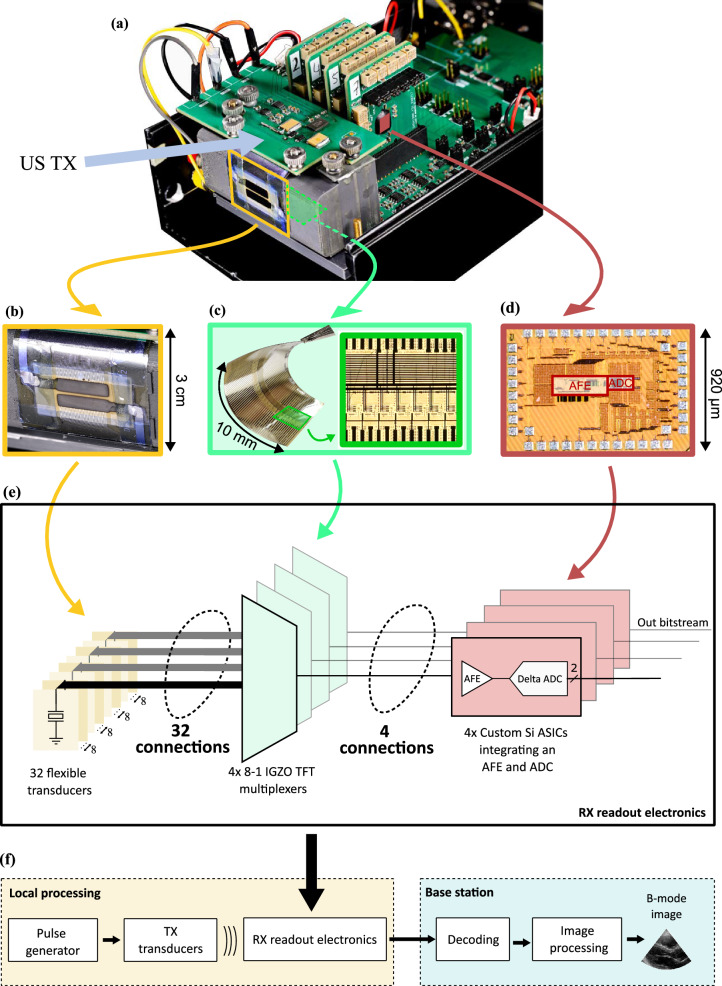
Prototype of ultrasound system. **a** Picture of the ultrasound prototype. **b** Close-up of the ultrasound transducers. **c** 4×8-1 multiplexer foil, shown when flexed, and a close-up of the multiplexer circuitry. **d** Micrograph of the silicon die, integrating an AFE and an ADC. **e** Block diagram of the receiver (RX) electronics. **f** Bock diagram of the complete implemented system.

In this work, we demonstrate a first step towards realizing this vision. A prototype (Fig. 1) is built using a US transducer array with 32 PillarWave^TM^ transducer elements, which are manufactured on a plastic foil using thermal embossing of a piezoelectric polymer^32^. The 32 transducers are connected to four 8-1 pitch-matched multiplexers, built on a separate foil using a-IGZO TFTs^33^. Thanks to these flexible TFT circuits, only four silicon front-ends, which are designed in a commercially available 65 nm Si-CMOS process, are required to interface all 32 transducers.

To tackle the front-end power challenge, a custom solution is proposed that adapts its power consumption based on the expected signal strength. Furthermore, we envision that image processing must be done in a base station, as shown in Fig. 1f. Indeed, this is a digitally intensive process which requires a high power consumption, and is likely not feasible on the patch. To alleviate the data communication problem described in the third challenge, the use of a specialized log-delta Analog to Digital Converter (ADC) is proposed. This method effectively decreases the volume of data that needs to be sent, making the system more efficient and reducing the energy needs of the data transmitter.

## Results

### Transducer and TFT multiplexer foils

**Fig. 2:**
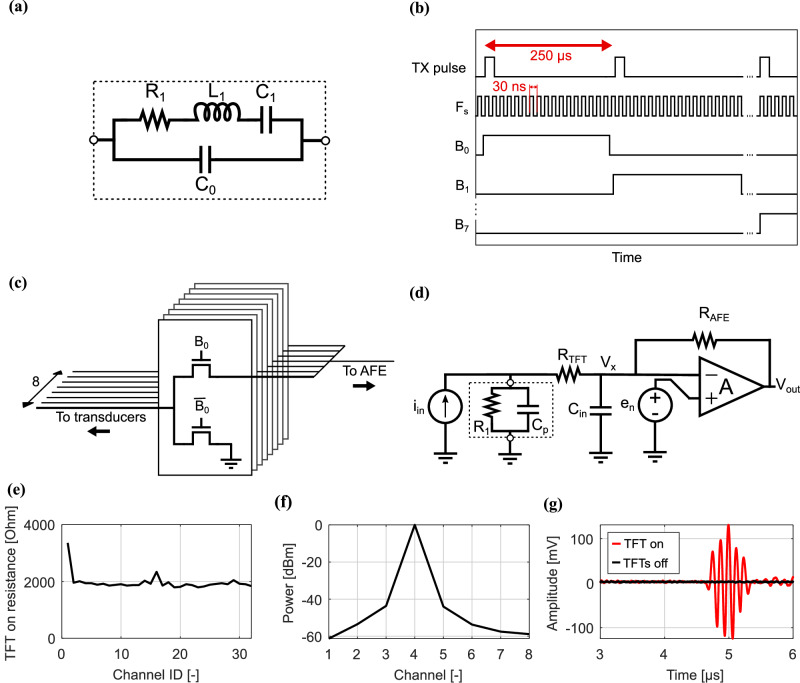
a-IGZO TFT multiplexer electrical modeling and performance. **a** Van Dyke model of the US receiver impedance. **b** Timing diagram of the multiplexer control signals. **c** Schematic of a single 8-inputs, 1-output multiplexer. This structure is repeated 4 times in our US system prototype. **d** Simplified electrical model of the transducer, TFT and TIA. **e** Distribution of the measured TFT switch on-resistance across the different channels. **f** Measured power cross-coupling between different channels of the TFT multiplexer (when exciting channel 4). **g** Recorded MUX output with one switch is closed (red) and all switches open (black).

The US transducer elements used in this prototype have a 180-µm pitch. The transducer array consists of separate transmit and receive apertures with 64 and 32 elements, respectively. In this way high voltage on the receiver (RX) multiplexer is avoided. The center frequency of the transducers is 8.2 MHz, and the frequency bandwidth is 78% at -6dB. The manufacturing process is described in ref. ^32^. The Butterworth-Van Dyke model (Fig. 2a) is used for electrical modeling of the US receiver.

To reduce the number of silicon front-ends, the RX elements are multiplexed using a flexible a-IGZO TFT technology with a minimum feature size of 0.6 µm. The TFTs are manufactured by Pragmatic^33^. Given the speed limitations of these TFTs, dynamic switching between transducer elements within a single acquisition is not feasible with this a-IGZO technology, as it requires switching the TFTs faster than the sampling frequency (1/*F*_s_ ≈ 30 ns). Instead, a single RX transducer element is selected for each multiplexer per acquisition cycle, necessitating the generation of a new transmit (TX) pulse for each new acquisition (Fig. 2b).

Budgeting e.g. ~250 µs of measurement time to reach an imaging depth of 18.5 cm and allow for the echoes to die down, leads to 400 frames per second if plane wave compounding is used with 10 waves. To maintain a frame rate above 50 fps while minimizing system complexity, we employ an 8-to-1 multiplexer. While multiplexing reduces the number of interconnections, it increases the TX energy consumption by 8, as multiplexing requires a new transmit pulse each time that a new set of RX channels is selected via the multiplexers. Thus, multiplexing introduces a trade-off between wiring complexity and power efficiency, as discussed in detail in Supplementary Note 9.

The schematic of the multiplexer circuit is shown in Fig. 2c. Four 8-input multiplexers are used to control all 32 RX transducer elements. When an element is not selected, it is switched to ground, reducing crosstalk.

To provide further insight, the circuit shown in Fig. 2d is used, where the on resistance of the TFT is modeled as resistor *R*_TFT_. In the Butterworth-Van Dyke model the series inductance *L*_1_ and capacitance *C*_1_ are assumed to be in resonance. The capacitor *C*_p_ lumps together *C*_0_ (~100 fF in our case) and the parasitic capacitance before the TFT. The parasitic capacitance after the TFT is represented by *C*_in_. Due to the two-tier integration of the transducer and TFT array, *C*_p_ is relatively large (5 pF). If one would choose a voltage amplifier, due to the presence of *C*_p_ and *C*_in_, the bandwidth of the transfer function from *I*_in_ to *V*_x_ is only 80 kHz, leading to an attenuation of 160x at 12 MHz. In contrast, choosing a TIA as done in our implementation, a virtual ground is created at the node *V*_x_, minimizing the voltage change across the parasitic capacitances and improving the bandwidth.

The performance of this circuit is sensitive to the parasitic capacitances *C*_p_ and *C*_in_. Indeed, there is a linear relationship between C_in_ and the input-referred noise density^34^, thus *C*_in_ should be minimized. Additionally, the virtual ground created by amplifier A causes *C*_TFT_ and *C*_p_ to form a low-pass filter. Due to the two-tier integration of the transducer and TFT array, the parasitic capacitance *C*_p_ is relatively large (5 pF). To maintain a cut-off frequency above 16 MHz, the TFT on-resistance must remain below 2 kΩ. This ensures that signal attenuation stays within 19% at the edge of the transducer bandwidth (11.4 MHz). Measurements (Fig. 2e) show that the TFT on-resistance is indeed approximately 2 kΩ, except for two channels: channel 1 has a resistance of 3.3 kΩ and channel 16 of 2.3 kΩ). This is expected to remain true even when bending the TFTs, thanks to the flexibility of a-IGZO TFTs, which change mobility by only 3% for a bending radius of 2 mm^35^.

To assess crosstalk in one 8-input multiplexer, a 200 mV_pp_ signal was applied to the input of channel 4, and the power coupled to the other channels was measured. The results (Fig. 2f), indicate that the crosstalk between adjacent channels in the multiplexer is around -45 dB, decreasing to -60 dB for more distant channels. The element-to-element crosstalk of the transducer array used in this work is −31 dB^32^. Therefore, the multiplexer does not contribute significantly to the overall system crosstalk.

To further validate multiplexer operation, Fig. 2g shows the output obtained when the TFT is turned on and off.

In conclusion, we have demonstrated the viability of using a-IGZO TFT switches to build a multiplexer in a two-tier on-foil integration with US transducers. Noise, bandwidth and crosstalk performance enable a first imaging demonstration, but we expect that tighter integration will ensure further improvements.

### AFE implementation

**Fig. 3:**
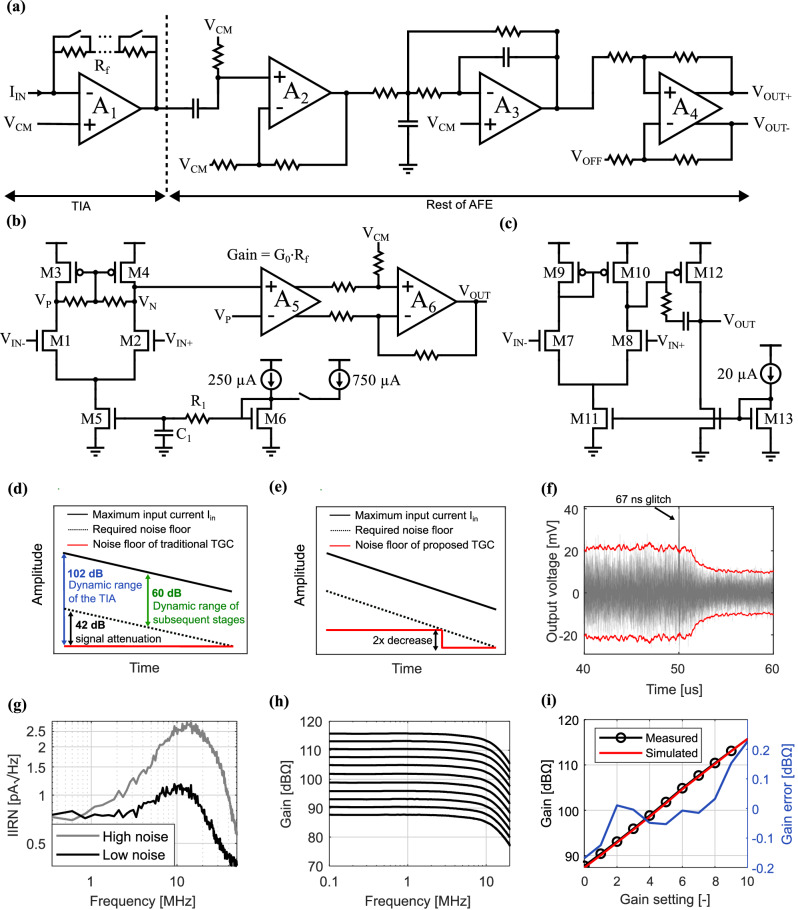
AFE electrical performance. **a** Circuit implementation of the AFE. The gain of the TIA is set by *R*_f_. **b** Circuit implementation of *A*_1,_ the gain of *A*_5_ is adjusted when *R*_f_ is changed to maintain stability as explained in the Supplementary Information**. c** Architecture of the circuit used to implement *A*_2_, *A*_3_ and *A*_6_. **d** Attenuation of ultrasound signal, required noise floor and noise floor of traditional implementations. **e** Noise floor of proposed TGC. **f** Transient noise signal of 40 different measurements and 3σ indication. **g** Frequency spectrum of the AFE in low and high noise mode. **h** Signal transfer function of the AFE with different gain settings. **i** Comparison of the measured and simulated gain of the TGC.

Figure 3a provides an overview of the circuit implementation of the CMOS analog front-end (AFE). The circuit implementations of A_1_ and A_2_/A_3_/A_6_ are shown in Fig. 3b, c, respectively. Among the AFE components, the TIA is the most power-hungry due to the low noise floor required, as is common in US systems^36^. Therefore, the rest of this section focuses on the design of this block.

In ultrasound imaging, an ultrasonic pulse excites a medium. As the acoustic wave propagates through the medium, absorption leads to a gradual attenuation of the signal (black line Fig. 3d). Due to this attenuation, earlier echoes (reflections from nearby objects) generally have a higher amplitude than later ones (from objects farther away). To counteract this loss of amplitude over time, ultrasound systems typically apply Time Gain Compensation (TGC), which increases signal amplification over time. In this way, echoes from objects farther away are amplified more, making the signal strength from all distances more uniform. For typical tissues the attenuation is 0.7 dB/(MHz·cm)^32,37^, which leads to an attenuation of at least 42 dB for a round-trip propagation till 4 cm depth, using a 7.5 MHz excitation signal. The actual attenuation will be even larger when considering reflection and spreading losses.

Implementing TGC reduces the dynamic range requirements of subsequent stages. To take advantage of this reduction early in the signal chain, the TGC is incorporated here into the TIA. The TIA gain is thus adjusted in discrete steps, using a variable resistor created with a stepped resistor network (Fig. 3a). Consequently, later stages need to handle only the reduced dynamic range indicated by the green arrow in Fig. 3d. However, the TIA itself must still accommodate the full dynamic range of the signal (blue arrow). This necessitates a noise floor of the TIA that is 102 dB below the maximum input current, assuming a 60 dB range of the subsequent stages (Fig. 3d). The noise performance of the TIA is determined by its thermal noise, which is inversely proportional to the square of the current through transistors M_1_ and M_2_ (Fig. 3b) when operated in subthreshold. Achieving a low noise floor thus requires a high current through these transistors, making the TIA extremely power hungry. However, since a low noise floor is only necessary near the end of the US measurement, we propose here to dynamically increase the amplifier power consumption by 4x only when weak echoes from far away must be detected (Fig. 3e). Figure 3f shows 40 transient responses of the AFE output when switching between the two modes. The red 3σ line indicates that the noise indeed decreases by approximately a factor of 2. A brief glitch, lasting around 67 ns is present. This is due to parasitic coupling in the measurement setup and can be reproduced in simulations when adding a 10 fF capacitor between the “low noise” enable signal and the signal input.

Figure 3g shows the measured AFE input-referred noise spectrum with and without the low-noise mode enabled. The total in-band noise is 12.9 nA_rms_ and 5.5 nA_rms_, respectively. The AFE’s measured transfer function is shown in Fig. 3h, and confirms a bandwidth of 12 MHz across all gain settings. The linearity of the gain settings is observed in Fig. 3i, which shows a maximum error of only 0.2 dBΩ compared to the ideal case.

### Log-delta ADC implementation

**Fig. 4:**
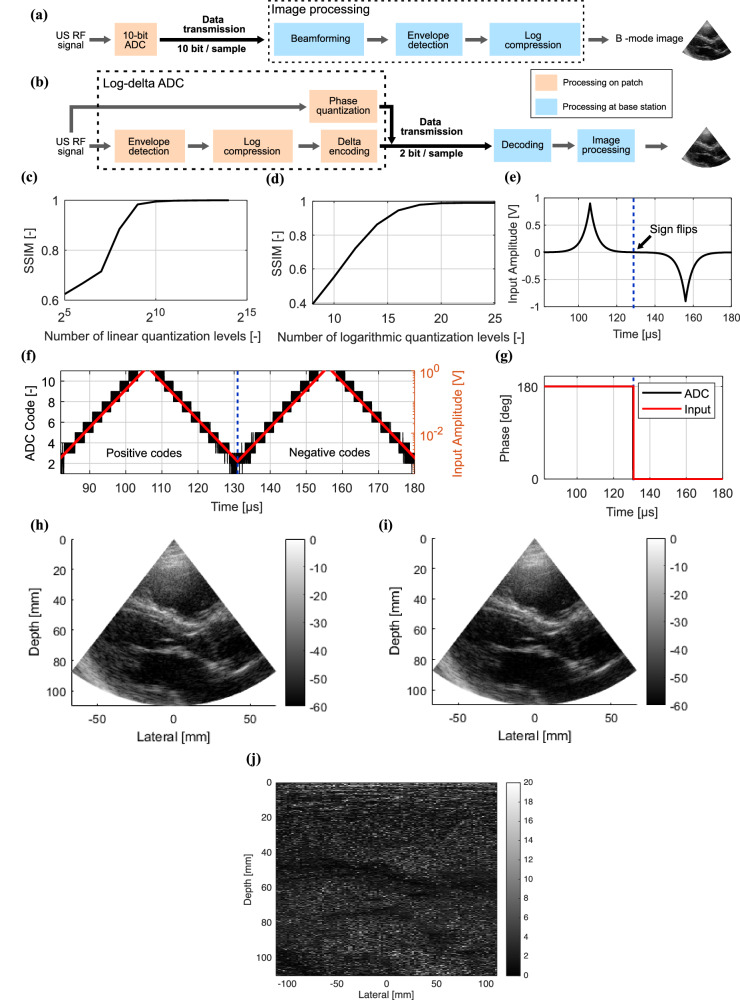
ADC concept and measurements. **a**, **b** Conventional (**a**) and proposed (**b**) approach for processing ultrasound signals. **c**, **d** Structural Similarity (SSIM) comparison for different quantization strategies: conventional linearly spaced quantization levels (**c**) and logarithmically spaced levels (**d**). **e** Exponential input signal used to validate the ADC functionality. **f**, **g** Transient response of the ADC: magnitude (**f**) and phase (**g**)**. h**–**j** B-mode images obtained using conventional processing of the original RF data (**h**), employing the measured output from the proposed ADC (**i**), and the difference between the two images (**j**).

A typical processing chain for converting ultrasound signals into an image is shown in Fig. 4a. The ultrasound RF signal is digitized using a 10–14 bit ADC. Once digitized, the data are transmitted to the digital processor where the signals undergo beamforming, envelope detection and logarithmic compression. Simulations with measured data from a human heart dataset^38,39^ show that a 10-bit ADC maintains Structural Similarity Index Measure (SSIM) greater than 0.95 with the original image, providing a baseline for comparing our data compression approach (Fig. 4c).

The 48 Tb/s output data rate discussed in the Introduction refers to the instantaneous data rate during ultrasound signal acquisition, assuming that all 1.5 × 10⁵ transducer elements are sampled in parallel at 32 MS/s with a 10-bit ADC. When considering a 250 µs acquisition time per frame, 50 frames per second and buffering data on the patch, the average required data throughput would be approximately 60 Gb/s. Even at this reduced average rate, the burden on wireless data transmission and power consumption remains substantial, justifying the need for on-chip data compression techniques.

There exist several channel reduction techniques, such as sparse arrays^40–44^, row-column arrays^45–47^ and micro-beamforming^48^. The method we propose here is complementary to those techniques, reducing the data from a single channel. Even though compressed sensing^49–51^ can also reduce single-channel data, it requires intensive digital processing.

To minimize processing overhead, we propose delta modulation^52,53^ as a power-efficient and hardware minimalistic approach for data compression. In 1-bit delta modulation, each signal sample is compared to the previous one, transmitting only the difference: “1” if the current sample is larger, and “0” otherwise. This is an effective technique for data reduction if the rate of change is much slower than the sample rate. However, ultrasound RF data often show significant amplitude variation between consecutive samples because the sampling frequency is typically only four times the center frequency of the transducer^54–56^. This means that each sample represents a 90° phase shift of the high-frequency RF signal, causing rapid changes in amplitude between samples. In the human heart dataset^38,39^_,_ the maximum difference between consecutive 10-bit quantized samples reaches up to 60 codes out of 1024. Thus, a direct delta encoding of the raw US RF signal would require 6 bits. Applying delta encoding on the raw RF signal would thus reduce the data by only 40%.

To further reduce the amount of data, and only use one bit per sample to encode the delta, we propose two techniques. Instead of using many uniformly spaced quantization levels, only a few logarithmically spaced quantization levels are used (Fig. 4b): this allocates more levels to small signal amplitudes and fewer levels to large signal amplitudes. Although logarithmic compression is traditionally applied after beamforming, our simulations show that applying it before beamforming keeps the SSIM greater than 0.95 when using more than 20 quantization levels (Fig. 4d). Moreover, we apply envelope detection, which smooths out the signal and reduces its variation. Applying envelope detection before beamforming distorts the B-mode image because phase information is essential for the beamforming algorithm. To overcome this, 1-bit phase quantization is used to encode the phase of the RF data (i.e., sign detection). The combination of these two techniques (Fig. 4b) reduces the amplitude change between successive samples, making it possible to apply delta encoding and keep a SSIM greater than 0.95, using the same sampling rate of typical US systems (4x the center frequency) and only two bits per sample.

To validate the ADC’s functionality, an exponential signal was applied to the circuit (Fig. 4e). Due to the logarithmic compression implemented by the ADC, this input produces a linear sweep of the 22 ADC codes, 11 for the positive, and 11 for the negative wave. The ADC toggles between the two nearest quantization levels, as depicted in Fig. 4f, which allows to encode the output with 1 bit. Additionally, Fig. 4g confirms the accurate detection of the signal sign by the ADC, which is encoded with a second bit.

To further validate our digitization approach, ultrasound signals taken from a clinical dataset^38^ were replayed through an arbitrary waveform generator and fed to the proposed ADC. The images generated using the conventional and proposed approach are shown in Fig. 4h, i, respectively.

A comparison between Fig. 4h, i shows that the proposed approach preserves the major image features. Additionally, the speckle pattern remains consistent in both distribution and size. This observation is further supported by the difference image shown in Fig. 4j, where the differences appear random and relatively evenly distributed. The largest differences are observed in regions with low signal levels. If such differences are considered undesirable, increasing the number of quantization levels could reduce them, though this would come at the cost of a reduced compression rate.

By encoding the ultrasound RF data with only two bits, instead of the 10 bits used in the conventional approach (Fig. 4a), a fivefold reduction in data transmission is achieved. This lower data volume, directly translates in significant power savings during transmission, extending battery lifetime.

### System validation

**Fig. 5:**
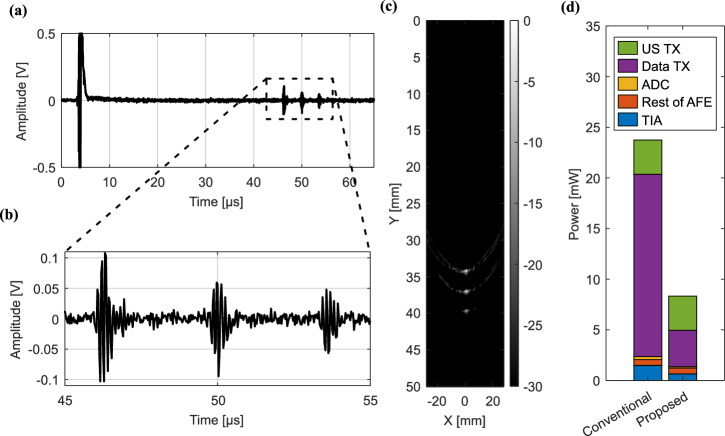
System performance. **a** Full transient ultrasound echo response from plane wave transmission (obtained decoding the log-delta ADC output). **b** Zoomed-in view of the reflections from the copper wires. **c** B-mode image captured using plane-wave imaging. **d** Power breakdown comparing the power consumption per channel with a conventional implementation and with the proposed techniques.

To validate the system depicted in Fig. 1e, f performing US imaging, the transducers are immersed in a water tank. Three 300-µm-thick copper wires are placed at a depth of 34, 37 and 40 mm, respectively. All TX transducer elements are excited simultaneously with five 200 V_pp_ pulses, generating a single plane wave. Figure 5a, b shows the ADC output after decoding (see the Methods section, paragraph ‘US system’, for additional details) when one transducer element is selected. A large pulse can be seen during TX excitation, due to electrical coupling between the TX and readout circuitry. After about 45 µs three pulses corresponding to the reflections of the copper wires are observed. Figure 5c shows the image obtained after performing 10 averages, and scaling the image with a 30 dB dynamic range from the brightest point. Due to the TX plane wave imaging, sidelobes are visible. Still, the three wires are clearly identifiable. The brightness of the first copper wire (peak signal) is -6 dB, and the background noise level in its vicinity is approximately -46 dB, resulting in an SNR of 40 dB.

Figure 5d presents a breakdown of the power consumption per channel in the proposed prototype, and an estimate of the power consumption without power reduction techniques. For ‘US TX’ the power for the proposed and conventional approach is the same: it is the power consumed by the ultrasound transmit circuitry divided by 64 (the number of TX elements).

The power consumed by the chip I/O buffers to send the output data to the FPGA using a wire bus is reported as ‘Data TX’. The power that would be required for wireless transmission in an US patch to the base station will be likely much higher than the consumption observed in this work using a wired connection. Therefore, for wireless transmission, data compression becomes even more important.

For the ADC, the lowest-power 10 bit ADC with a comparable sampling speed found in the literature was used as a ref. ^57^. The observed power improvement in our implementation is primarily attributed to the comparator, which performs only three comparisons per sample instead of ten.

For ‘Rest of AFE,’ the power consumption would remain unchanged. For a conventional TIA implementation, it is assumed that the circuit continuously operates in ‘low-noise’ mode. In our approach, the ‘low-noise’ mode is active only 20% of the time. This provides a 2.3x reduction in TIA power consumption. When these savings are compared to the overall power consumption of the AFE, the result is a reduction of 42%. When comparing the estimated power consumption without (left column) and with (right column) all power reduction techniques, the total power consumption improves by a factor 2.86.

## Discussion

In this work, we have taken a step toward realizing the vision of a fully flexible ultrasound patch with a massive number of transducer elements. Specifically, we addressed three grand challenges: the interconnection of transducers and electronics, high front-end power consumption, and data communication demands.

To address the challenge of interconnections, we demonstrated the integration of flexible ultrasound transducers with flexible a-IGZO TFT multiplexers. This approach reduced the number of interconnects to the silicon front-end chips by a factor 8, showing the viability of using TFT technology to manage the large-scale integration required for US flexible patches.

The second challenge of high front-end power consumption was tackled through the development of a power-adaptive front-end. This circuit dynamically adjusts its power consumption based on the expected signal strength, reducing the power needs by 42%.

To address the challenge of data communication demands, we incorporated a novel log-delta ADC. This ADC achieved a 5-fold reduction in needed data throughput, keeping the image quality. This approach reduces the power consumption required for data transmission.

These advances bring us a step closer to developing scalable, wearable ultrasound patches for continuous at-home monitoring. Future research will focus on expanding these advances towards the ultimate vision of flexible ultrasound patches.

Although the transducers and MUX are fabricated on flexible substrates, and using devices that are known to maintain their functionality while bent^26,32^, their behavior under mechanical deformation was not assessed in this work. Further studies are required to evaluate possible image degradation, the mechanical reliability under bending, and how to cope with e.g. element alignment for beamforming while using a flexible substrate, which are critical considerations for future wearable ultrasound applications.

A key next step is to scale from a linear to a 2D US array. Our results indicate that static selection of RX transducer elements using a-IGZO TFTs is feasible. We envision leveraging this capability to enable the selection of smaller subarrays (e.g., 8×8 elements) within a large 2D matrix, thereby managing the interconnect burden while enabling more complex imaging strategies.

While the current TFT technology does not support high-voltage transmission (TX) multiplexing, this is a technological limitation rather than a fundamental one. Future improvements in a-IGZO device fabrication, such as increasing gate dielectric thickness and optimizing switch area, could enable the use of high-voltage pulser ICs in combination with subarrays statically selected for transmission using TFTs. This hybrid approach would open the door to TX beamforming and full phased array imaging, bringing high-resolution, real-time 3D imaging closer to reality in a compact, integrated system.

## Methods

### Transducers

The ultrasound transducer foil is manufactured by TNO. Details on the manufacturing method can be found in ref. ^32^. The foil consists of two rows of flexible transducers, each containing 64 elements with a pitch of 180 μm and are connected to gold traces on the foil. One row was used for excitation, with all transducers pulsed simultaneously to generate a plane wave, while 32 transducers of the second row were used for signal readout. The flexible transducers were mounted onto a custom 3D-printed polylactic acid (PLA) adapter fabricated using a 3D printer (Ultimaker 3). Alignment markers were incorporated into the design to facilitate initial positioning.

The gold traces on the transducer foil were aligned to pads with the same pitch on the printed circuit board (PCB) under a microscope for precise positioning. The 3D-printed piece, along with the connected transducer traces, was secured to the PCB using M3 screws, to establish intimate electrical contact. A 200 µm thick thermoplastic polyurethane (TPU) layer was inserted between the transducer foil and the PLA adapter to compensate for surface unevenness. To prevent oxidation and ensure stable electrical connections, the PCB pads were coated with an electroless nickel immersion gold (ENIG) finish.

Measurements were conducted in a water-filled box at room temperature. The 64 TX transducers were excited using a 5-cycle pulse train. The pulses have a duty cycle of 50% and a period of 132 ns. 3.3 V pulses were generated by an arbitrary waveform generator (33500B, Keysight) and amplified to 200 V_pp_ using the high-voltage amplifier chips (HV7360GA-G, Microchip Technology), powered by ±100 V supplies from Delta Elektronika SM400-AR-4.

### A-IGZO multiplexer foil

The a-IGZO foil is fabricated by Pragmatic^33^. It was clamped to the PCB using a 3D-printed PLA piece, following the same process as for the transducers. The multiplexer foil implements four 8-1 multiplexers, and is thus able to accommodate 32 transducer elements. Due to the sensitivity of a-IGZO thin-film transistors (TFTs) to electrostatic discharge (ESD), protective measures were implemented. The foil included reverse-biased diodes, created using diode-connected transistors, for local ESD protection, while ESD-suppressing diodes were integrated into the PCB for additional protection.

To prevent voltage buildup during handling, all pads on the a-IGZO foil were shorted together. After alignment and secure mounting on the PCB, these shorted connections were manually severed to enable individual electrical connections.

### CMOS chip

The four CMOS chips, each containing one processing chain including AFE and ADC, were fabricated using a commercial 65 nm silicon process and wire-bonded to a separate PCB (daughter boards) for electrical characterization. The daughter boards were connected to the main system PCB via a PCIE connector, allowing modular testing in different setups. The PCB pads were coated with an ENIG surface finish to prevent oxidation.

A 32 MHz clock was applied to the CMOS input using a clock generator (CG635, Stanford Research), and bias currents were adjusted using a potentiometer. The AFE and ADC offsets were jointly tuned by observing the ADC output and adjusting *V*_off_ (Fig. 3a) using a potentiometer.

The power consumption of the CMOS chip is measured using a source meter (2450, Keithley). By separating the supply pins of the TIA, AFE and I/O buffers, the power consumption of each block could be characterized.

### SSIM simulations

To assess the effect of amplitude quantization on ultrasound image quality, we performed a series of simulations and evaluated the Structural Similarity Index Measure (SSIM). These simulations were carried out using MATLAB and the k-Wave toolbox^58^. The dataset used for these simulations was acquired from in vivo human heart measurements^36,37^.

SSIM was calculated using the standard equation^59^:

1 $${SSIM}=\frac{2{\mu }_{x}{\mu }_{y}\cdot 2{\sigma }_{{xy}}}{({\mu }_{x}^{2}+{\mu }_{y}^{2})({\sigma }_{x}^{2}+{\sigma }_{y}^{2})}$$

where ${\mu }_{x}$ and ${\mu }_{y}$ are the mean intensities of images X and Y, ${\sigma }_{x}^{2}$ and ${\sigma }_{Y}^{2}$ their variances and ${\sigma }_{{xy}}$ is the covariance. The raw RF ultrasound data was first normalized between 0 and 1 prior to quantization. Three different quantization strategies were then explored, as discussed in the subsections here below.

1. Linear quantization. The normalized RF data were uniformly quantized using a varying number of quantization levels, ranging from 2^5^ to 2^15^. Each sample was rounded to the nearest quantization level. Figure 4c summarizes the SSIM values obtained for each level. It shows that a minimum of 2^10^ linear quantization levels was required to maintain an SSIM above 0.95.
2. Delta quantization. Next we analyzed what would happen applying delta encoding to the data uniformly quantized using 2^10^ levels. In this case, it was found that the maximum difference between two consecutive samples can reach up to 60 codes out of the 2^10^ quantization levels. Therefore, direct delta encoding of the raw US RF signal would require 6 bits.
3. Log delta quantization. Finally, we analyzed how many logarithmically spaced quantization levels would be needed to maintain an SSIM greater than 0.95. The logarithmic quantization was designed to be symmetric around the mean. The SSIM is greater than 0.95 when using more than 20 quantization levels (Fig. 4d).

### A-IGZO multiplexer measurement setup

All electrical measurements made to characterize the multiplexer (Fig. 2) were performed using a PCB containing a transimpedance amplifier (TIA) using an ADA4817 operational amplifier with a 20 kΩ feedback resistor and a 0.4 pF feedback capacitor to ensure stability. An additional non-inverting amplifier (15× gain) was cascaded to the TIA. The multiplexer channels were tested using a 28 mV_pp_ signal applied using SP100 probes. The output was read out using a spectrum analyzer (HP3588A, Keysight) during both coupling and resistance measurements. Time-domain measurements (Fig. 2g) were captured using an oscilloscope (MSOX6004A, Keysight).

### AFE measurement setup

For Fig. 3e, the TIA was set to its highest gain configuration, switching from high-noise to low-noise mode. The AFE output signal was extracted using an operational amplifier (MIC920YC5, Microchip Technology) connected in buffer configuration and recorded on an oscilloscope (MSOX6004A, Keysight). Frequency-domain measurements were conducted using a spectrum analyzer (HP3588A, Keysight), with a 2 mV_pp_ input signal applied to the TIA through a 10 kΩ resistor.

### ADC measurement setup

ADC functionality was validated by applying an exponential input signal from an arbitrary waveform generator (33500B, Keysight). The ADC output was recorded with the oscilloscope (MSOX6004A, Keysight). Figure 4f shows the processed data after the signal supplied by the arbitrary waveform generator was digitized by the ADC.

### US system measurement setup

The setup used to perform the imaging reported in Fig. 5 is shown in Supplementary Fig. 1. It includes all blocks inside the box “Local Processing” in Fig. 1f. The output of the ADC is log compressed. This compression is removed in the base station (box “Decoding” in Fig. 1g) using

2 $$D[i]={sign}[i]\cdot {2}^{{Y}_{{ADC}}[i]}$$

where *D[i]* is the uncompressed signal, *sign[i]* the sign of sample *i* and *Y*_*ADC*_ the output code of the ADC. The results were averaged 10 times to improve the signal-to-noise ratio. A Gaussian filter with a center frequency of 8 MHz and a 50% bandwidth was applied to the data. Delay-and-sum beamforming was employed, followed by a Hilbert transform for envelope detection. Finally, logarithmic compression was applied to optimize the dynamic range of the resulting signal. The CMOS chips were controlled using an FPGA (Genesys 2, Digilent), with the PCB connected to the FPGA via an FPGA Mezzanine Card (FPC) High Pin Connector (HPC). The ADC operated at 32 MHz, and the data was read out through the FPGA. The FPGA buffered the ADC data and stored it in BRAM. Data acquisition was managed through a UART connection to a computer, where the data was transferred to Matlab for further processing.

### Imaging setup

Supplementary Fig. 1 shows the experimental setup used for ultrasound imaging. At the bottom of the setup is a water-filled box containing the copper wire phantom, which serves as the imaging target. Above the box, the transducers are mounted on a custom 3D-printed holder that is securely attached to the printed circuit board (PCB). Electric signals generated by the 32 receiving transducers pass through a multiplexer foil, which reduces the 32 connections to 4. The multiplexer is obscured in the picture by the 3D-printed holder.

These four outputs are then processed by four custom silicon chips mounted on the PCB, which integrate the AFE and ADC. The digitized signals are sent to the FPGA board for temporary storage. After a measurement is completed, the data is transmitted from the FPGA to the PC via a UART interface for further analysis.

Supplementary Fig. 2 shows a planarized overview with photos and PCB outlines of the US transducers, a-IGZO multiplexer, Si ASIC and the rigid PCB connecting the 3 components together. In this prototype, only half of the receiving transducers are used. To establish contact between the PCB traces and the transducer and between the PCB traces and the IGZO multiplexer, metal contacts on the different pieces are pressed together by pushing the foils against the PCB using suitable 3D printed pieces. The Si ASIC is bonded on a small separate daughter board to allow for separate electrical characterization. It is connected to the main PCB via a PCIE connector, schematically shown in the picture.

Supplementary Fig. 3 shows a closeup of the a-IGZO multiplexer foil. The bottom connections go to the US transducers. Between each trace going to a transducer element is a trace connected to the reference voltage *V*_cm,_ to provide additional shielding. The top connections (*V*_o1_, *V*_o2_, *V*_o3_ and *V*_o4_) go to the Si ASIC. Each connection is surrounded by a trace connected to VDD and VSS to provide additional shielding. The other traces are left floating and are added to help with the alignment of the foil to the PCB.

## Supplementary information

supplementary_final_clean

## Data Availability

The datasets generated and/or analyzed during the current study are available from the corresponding author on reasonable request.
