# Supplementary material for: An ultrasound imaging system exploiting transducers and multiplexers on a flexible substrate together with a log-delta CMOS ADC
Source: Npj Flex Electron. 2025 Oct 13;9(1):104. doi: 10.1038/s41528-025-00478-5 (PMC12518120; doi:10.1038/s41528-025-00478-5)
Supplement: Supplementary file 1 — supplementary_final_clean [file 41528_2025_478_MOESM1_ESM.docx]

Supplementary information

**An Ultrasound Imaging System Exploiting Transducers and Multiplexers on a Flexible Substrate together with a Log-Delta CMOS ADC**

Martijn Timmermans^1^, Kyle van Oosterhout^1^, Marco Fattori^1^, Paul van Neer^2^,

Pieter Harpe^1^, Eugenio Cantatore^1^

^1^ Eindhoven University of Technology, Eindhoven, the Netherlands

^2^ Acoustics & Underwater Warfare, TNO, The Hague, The Netherlands

Email: m.w.timmermans@tue.nl


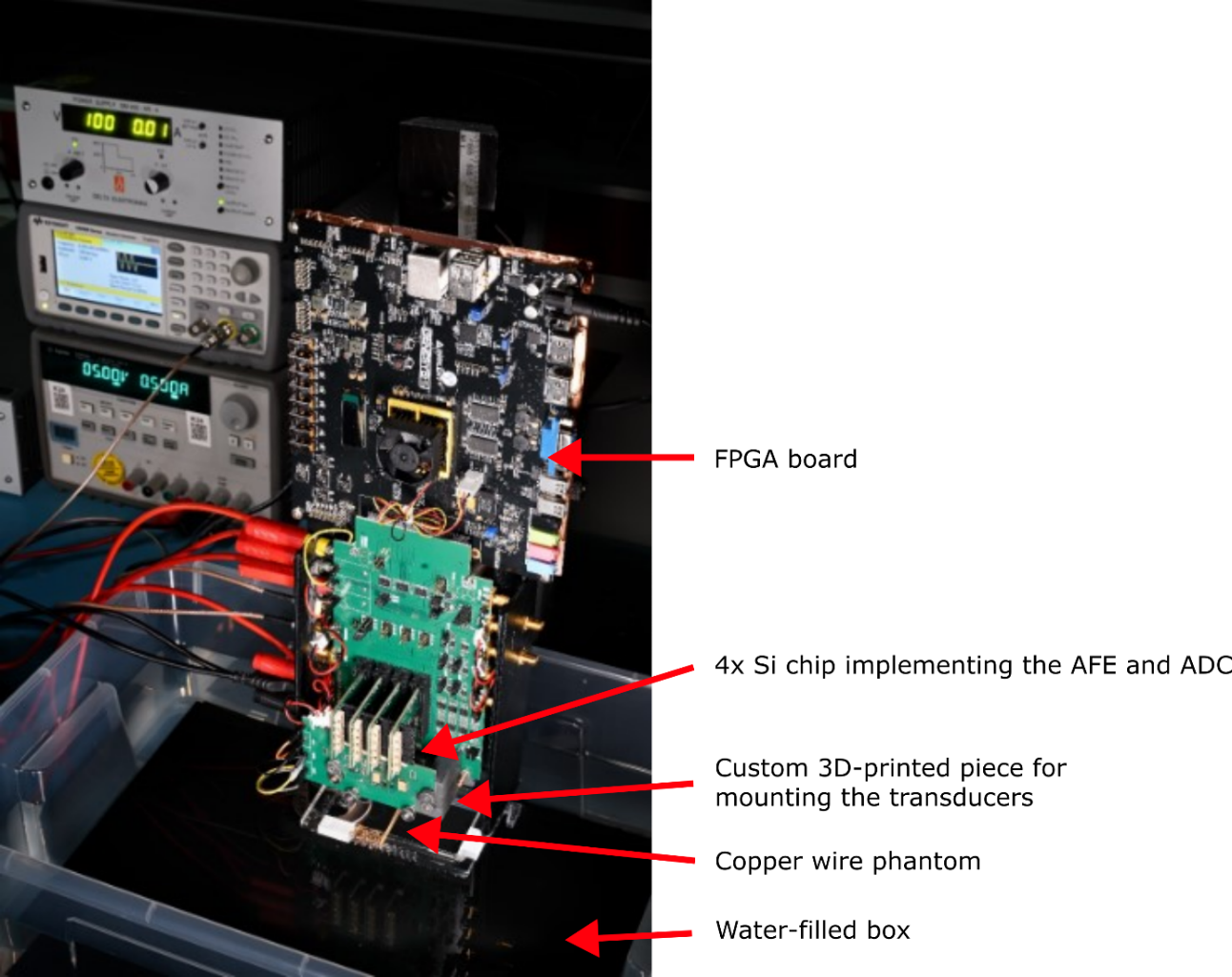


**Supplementary Fig. 1 | Experimental Imaging setup.**


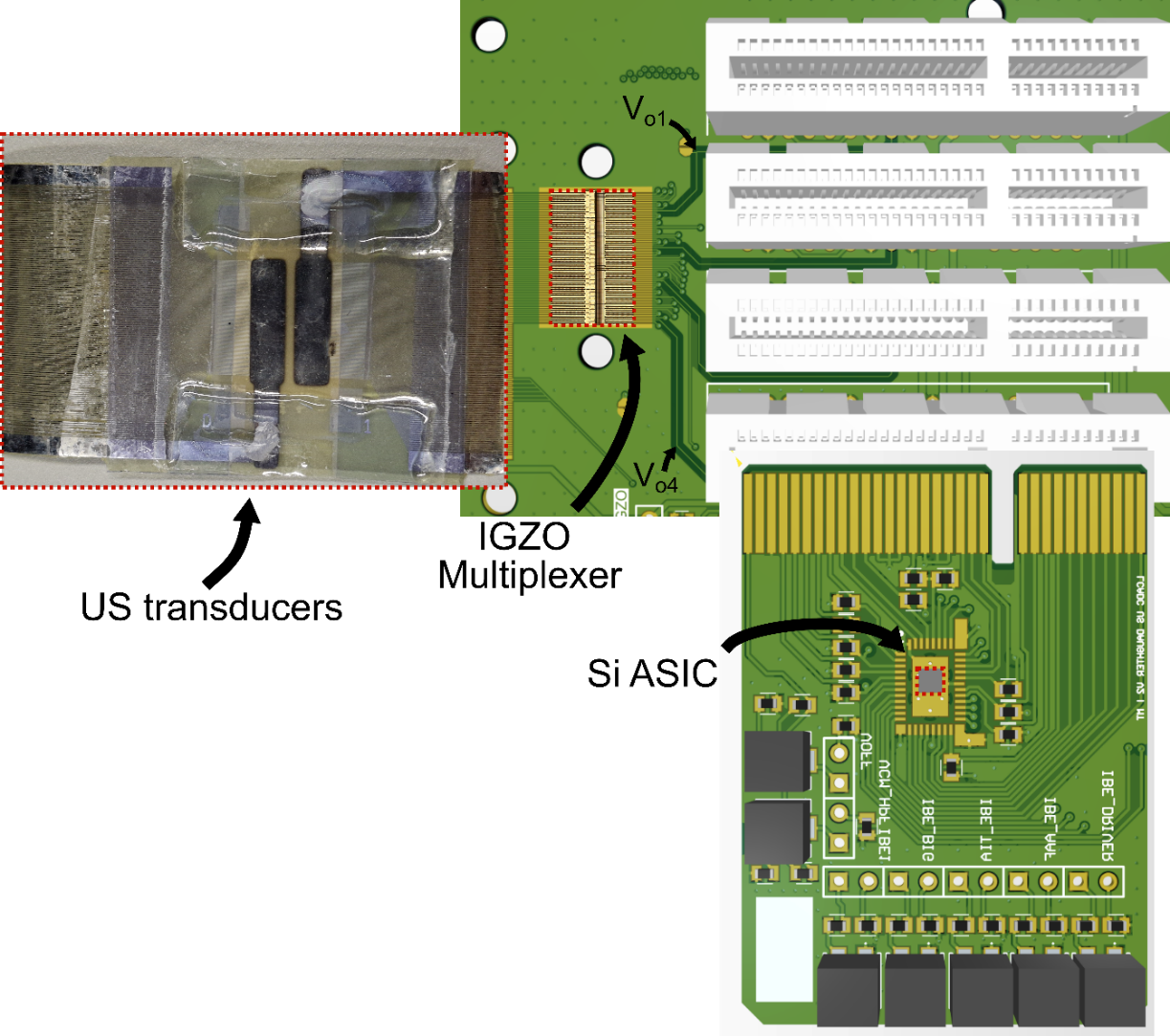


**Supplementary Fig. 2 | Planarized overview of the US transducers, a-IGZO multiplexer and Si ASIC**


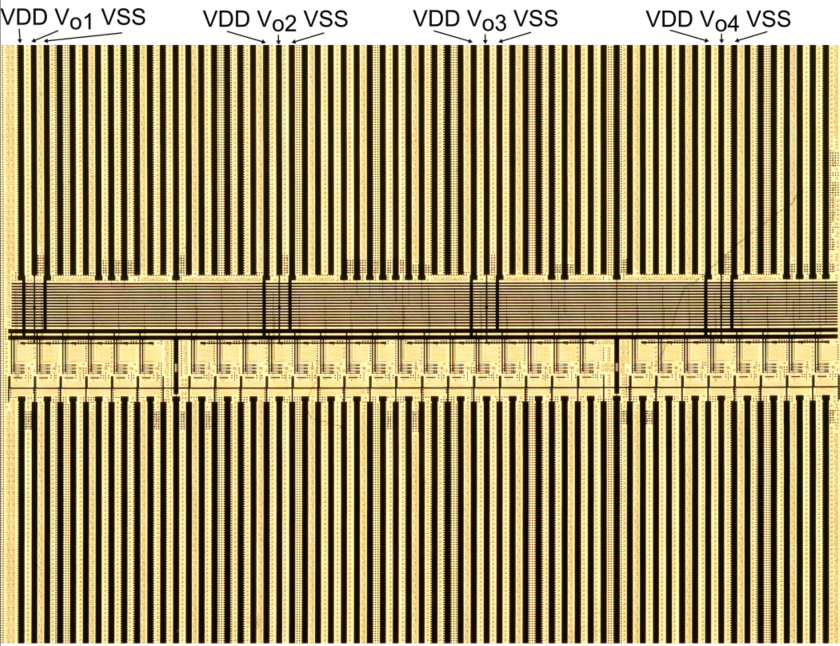


**Supplementary Fig. 3 | Close up of the a-IGZO TFT Multiplexer**


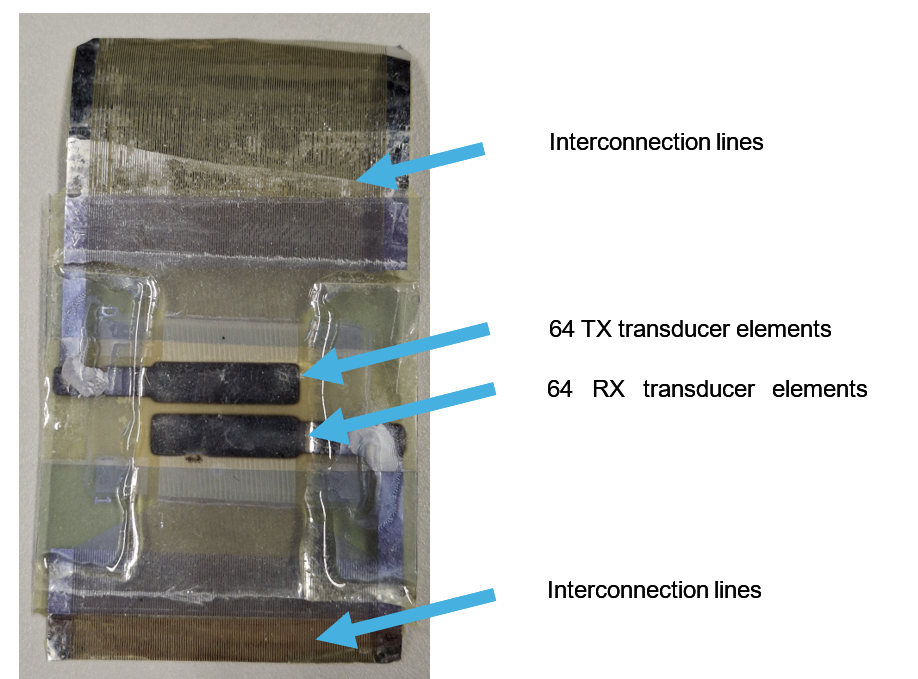


**Supplementary Fig. 4 | Close up of the ultrasound transducers**

**Supplementary Note 1.** **Scaling considerations for a-IGZO multiplexed ultrasound arrays.**

To address the challenge of scaling to very large 2D ultrasound arrays (~10⁵ elements), we evaluated three possible architectural approaches for integrating low-cost, large-area multiplexing technologies based on e.g. a-IGZO TFTs.

1. Dynamic Switching Scheme

Dynamic switching of the receiver transducers would be the most desirable approach for real-time imaging, allowing flexible element selection and beam steering. However, the high on-resistance (R_TFT_) of current a-IGZO TFTs imposes a critical limitation (Fig. 2d). To maintain 3τ settling for e.g. 8:1 multiplexing at a 32 MHz sampling frequency, the R_TFT_·C_p_ product must support a bandwidth of 768 MHz. Some improvement can be achieved with monolithic integration of transducers and TFT multiplexers, together with minimization of the interconnect length between the multiplexer and the Si ASICs. This could reduce the parasitic capacitance (C_p_) from the 5 pF measured in our system (based on hybrid integration of transducers, multiplexers and Si chips), to e.g. 2pF in a favourable scenario. Thus, R_TFT_ would still need to be reduced by approximately 19×.

R_TFT_ is inversely proportional to device parameters (W/L, oxide capacitance C_ox_, and carrier mobility μ) and to the gate voltage overdrive V_gs_-V_t_. Increasing W increases C_p_ too, providing no net speed benefit if the TFT capacitance dominates the total parasitics. Similarly, increasing C_ox_ lowers R_TFT_ but increases the TFT parasitic capacitances by the same factor. The overdrive voltage is limited by oxide breakdown, and the mobility is material-dependent. The best option is to decrease L with advances in technology, but this comes at increased production cost. Other flexible TFT options such as amorphous silicon (a-Si), zinc oxide (ZnO), and organic TFTs suffer from significantly lower mobility than a-IGZO, which translates to higher on-resistance for the same switch size, making them even less suitable for dynamic switching in high-frequency ultrasound systems. Low-temperature polycrystalline silicon (LTPS) offers roughly 5× better mobility than a-IGZO, potentially reducing on-resistance. However, LTPS devices typically suffer from much longer channel lengths, eliminating the mobility advantage in terms of R_TFT_. All in all, thus, the most feasible path to future dynamic switching of the receivers is in our view to scale down the length of the TFTs used for multiplexing, incurring in larger manufacturing costs.

1. Implementing the Front-End with TFTs

Another strategy is to integrate the entire front-end using TFTs. High transition frequencies (fₜ > 100 MHz) have been demonstrated in recent a-IGZO TFTs [7], indicating potential for fast signal processing. In this context, LTPS might become also attractive due to its higher carrier mobility. However, the low-noise requirements for ultrasound receivers demand high power, and TFTs are inherently less power-efficient than silicon. Consequently, this approach could result in high power consumption and is therefore less attractive.

1. Static Switching Scheme

Given the above constraints, we adopted a static switching scheme in this work. This approach allows us to reduce the amount of connections by 8x. Scaling further this way does not seem feasible without sacrificing the frame rate. Therefore, when scaling to larger 2D arrays, we think that static selection of a subset will be required. While this means that only a subset of elements can be read out at any given time, it offers advantages in terms of simplicity, power efficiency, and scalability.

In practical ultrasound systems, it is not necessary or desirable to read out all ~10⁵ elements simultaneously. Based on the current state of the art [3], the power consumption for a single-channel custom ASIC is 0.66 mW. Reading out all elements at once would thus require over 66 W, which is very difficult to conceive in portable or wearable systems. Instead, we propose using static switching to select a smaller subarray for each acquisition cycle, thereby significantly reducing instantaneous power and data bandwidth requirements. In this approach, only the sub-arrays of US transducers that are best placed to provide a meaningful image will be used..

Importantly, this approach does not limit imaging frame rate when scaling to large 2D arrays. High frame rates can still be achieved by sequentially selecting different subarrays across acquisition cycles. This allows the system to scan the entire US array over time and dynamically focus only on relevant regions of interest—aligning well with the goals of intelligent, sparse acquisition strategies in large-array imaging.

**Supplementary Note 2. Stabilizing the TIA**

The lowest power consumption would be achieved continuously adjusting the power consumption of A_1_ (Fig. 3a) to ensure that the noise floor of A_1_ follows the dashed line in Fig. 3d. However, this approach is not straightforward, as it would require a quadratic increase in current over time. Due to this quadratic relationship, most of the power is consumed at the latest moments in one acquisition, when smaller inputs are expected and lower noise is demanded. As a result, a good trade-off between circuit complexity and minimizing power consumption is found in the solution presented in Fig. 3e, where the current is increased by a factor of 4 in a single step. In contrast, a previous work [8] scaled the power by a factor of 2, due to stability concerns. We employed different techniques to maintain stability across all gain and noise settings, as described below.

To analyse the stability of the TIA, the circuit in Supplementary Fig. 5a is used. This circuit consists of an amplifier A_1_, a feedback resistor R_f_​, which sets the TIA gain, and a parasitic capacitance C. For this analysis, we assume that the amplifier has a single internal pole f_p1_, while the combination of R_f_​ and C creates another pole, referred to as the "input pole." We designed this input pole to be the dominant pole of the TIA to maintain stability even when the input capacitance is larger than expected. While an increased capacitance would reduce the TIA's bandwidth, it ensures that the operation remains stable.

Stability is analysed using the Bode plot of the transfer functions for A and the reciprocal feedback factor 1/β, as shown in Supplementary Fig. 5b. The unity gain frequency f_u_ is defined as the point where the lines for A and 1/β intersect. For stability, it is crucial that the amplifier's pole f_p1_​ is at a sufficiently higher frequency than f_u_. This ensures that the phase shift introduced by the amplifier's pole does not significantly affect the phase margin at the unity gain frequency, thereby maintaining stability.

The TIA must remain stable across all gain settings, which is controlled by varying R_f_. Supplementary Fig. 5b illustrates two scenarios: one with a higher resistor R_f_ and another with a lower resistor R_f_’. If the amplifier gain A remains unchanged (black solid line), the system becomes unstable when R_f_’ is selected, as the amplifier pole f_p1_​ occurs before the unity gain frequency. To prevent instability, the amplifier's gain A must be reduced (grey solid line) when R_f_​ decreases.

Assuming the input pole dominates the amplifier's pole, the TIA's bandwidth f_BW_​ can be calculated as:

$$\begin{aligned} f_{BW}=\frac{A}{2\pi R_{f}C} \#\left( S1 \right) \end{aligned}$$

This corresponds to the unity gain frequency of the feedback loop and can also be identified in the plot as the intersection of A and 1/β. To maintain a constant bandwidth, the amplifier gain A is scaled with R_f_​. As can be seen from Supplementary Fig. 5b, the worst-case scenario occurs when the highest R_f_​ is selected, determining the unity gain frequency f_GBW,A_​.

The circuit implementing the amplifier A in Supplementary Fig. 5a, is shown in Fig. 3b. Its gain is changed in in the second and third amplifier stage. Implementing a inverting feedback configuration in the second stage (A_5_) was not feasible due to its practical limitations. Indeed, choosing a small feedback resistor resulted in excessive loading of the first amplifier, reducing its gain. Conversely, selecting a large feedback resistor introduced significant thermal noise. To overcome this trade-off, we opted for using source degeneration as a technique to control the gain of A_5_. In the third amplifier stage (around A_6_), thanks to the gain of the second stage, it was possible to implement a inverting feedback configuration. By choosing suitable gain in A_5_ and A_6_, it is possible to maintain stability when changing R_f_.

Thanks to the TGC, the TIA gain changes by 30 dB along the full acquisition. However, only during the last 6 dB (highest gain settings), lowering of the noise floor is required. To achieve this, the current in the first stage of the TIA is increased by a factor of 4, reducing the input referred noise by a factor of 2. This means that the extra power is used only during the last 6/30 = 20% of the acquisition.


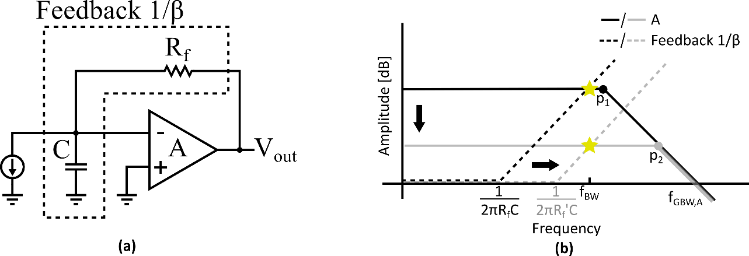


**Supplementary Fig. 5 TIA stability analysis | a**, circuit used for stability analysis. **b**, bode plot showing the transfer function of A and feedback 1/β.

**Supplementary Note 3. Dynamic biasing of the TIA**

The current of M_5_ (Fig. 3b) is increased in a single discrete step, which affects the differential output voltage of the first stage (V_p_ and V_n_) due to its finite common mode rejection. To mitigate this, three differential amplifiers are used in cascade to build A_1_, improving its total common mode rejection. The power overhead of the later stages is limited, thanks to the gain of the first stage which alleviates the noise constraints. A low-pass filter (R_1_, C_1_) is used to smoothen the change in the M_5_ current. This slows down the glitch in the differential output resulting from the change in bias current, and allows it to be filtered by the subsequent high-pass filter (Fig. 3a). Fig. 3f shows 40 transient response measurements when switching between the two modes. The red 3σ line indicates that the noise decreases by approximately a factor of 2. A brief glitch, lasting around 67 ns remains, due to parasitic coupling in the measurement setup.


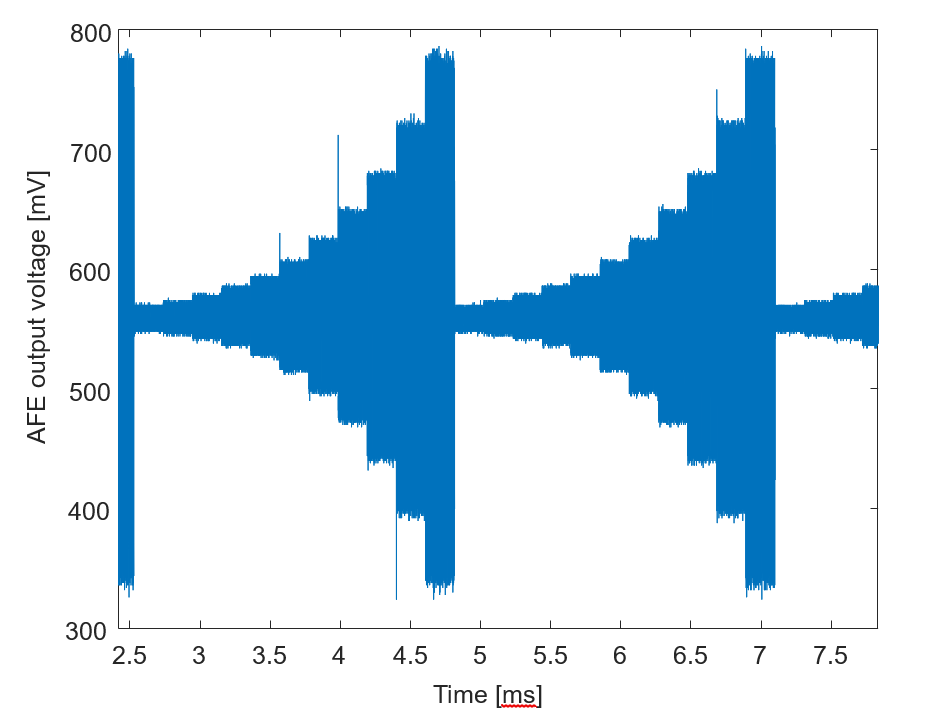


**Supplementary Fig. 6 Measured response of the AFE to a 500 nA_pp_ sinusoidal input while dynamically stepping through gain settings**

**Supplementary Note 4. Circuit implementation of the AFE**

The complete AFE circuit (Fig. 3a) consists of a transimpedance amplifier (TIA), a high-pass filter, an anti-aliasing filter [9], and a voltage buffer to drive the ADC. Amplifier A_2_, A_3_, and A_6_ all share the same architecture, which is shown in Fig. 3c.

The high-pass filter uses a non-inverting architecture and has a gain of 4. This gain helps to reduce the noise contribution from later stages and limits the input range that A_2_ needs to handle.

The low pass filter built around A_3_ uses the multiple feedback low pass filter architecture [9] and provides suitable antialiasing. It is designed with a bandwidth of 12 MHz and a Q factor of 0.7 to prevent signal peaking while providing a steep roll off.

The final block is a buffer which converts the single ended signal to a differential one and drives the ADC. Its implementation is shown in Supplementary Fig. 7. It uses a fully differential two stage amplifier with Miller compensation. The first stage has self-biased common mode via M_3_ and M_4_. The common mode of the second stage is set using an auxiliary low-gain amplifier to improve the output common mode robustness against PVT variations. The offset of the entire AFE can be tuned by V_OFF_ (Fig. 3)_._


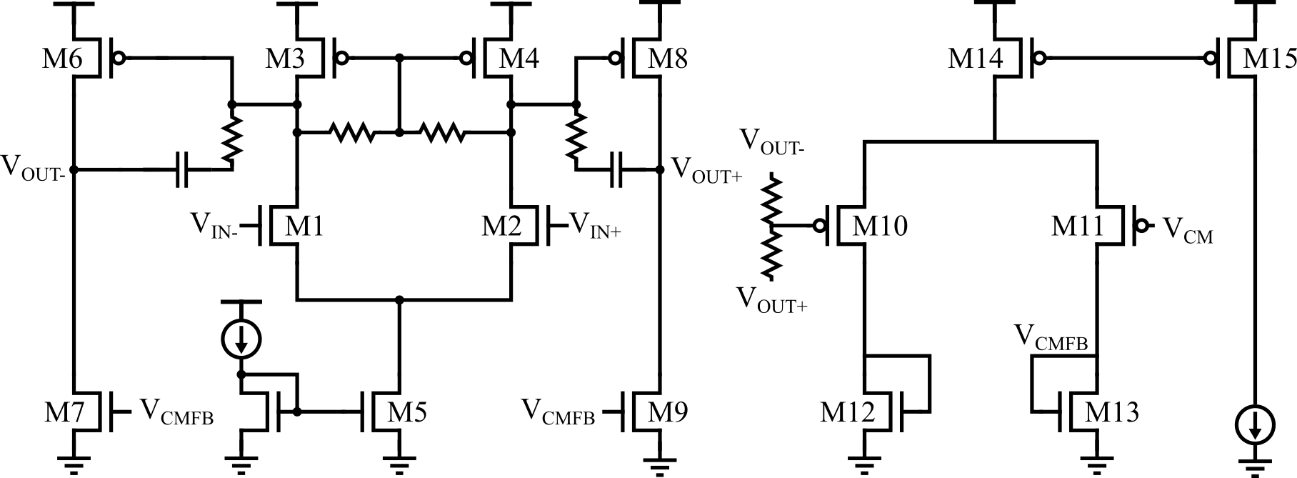
**Supplementary Fig. 7 Circuit implementation of A_4_**

**Supplementary Note 5. Circuit implementation of the log-delta ADC**

The circuit implementation of the ADC is shown in Supplementary Fig. 8. It employs a charge-sharing DAC which stores the level chosen to approximate the previous sample, and subtracts it from the input voltage of the present one. This operation generates a residual voltage that represents the difference between the present input sample and the previous reference level. A comparator determines the sign of this residue, to determine whether the present sample is larger or smaller than the previous reference level. Based on the comparator decision, the envelope of the signal is estimated. According to the result of the envelope estimation algorithm, a bit shift right or bit shift left operation is performed on the DAC code, effectively halving or doubling the reference level, achieving the logarithmically spaced quantization levels.

The comparator uses the low-power topology presented in [1].


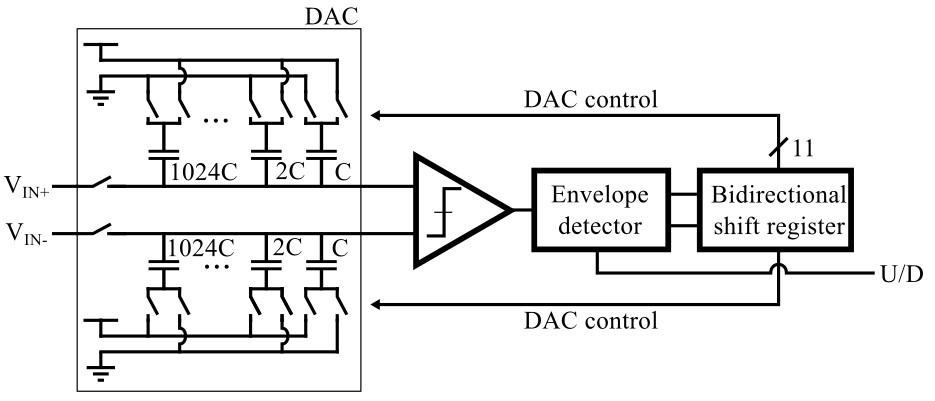


**Supplementary Fig. 8 ADC circuit implementation**

**Supplementary Note 6. Implementation of envelope detection**

The US reflections can typically be well described by a high frequency RF carrier (the US fundamental) which is amplitude modulated with a much lower frequency. Hence by performing envelope detection before the 1-bit delta modulation, we are able to remove the high frequency RF carrier, significantly reducing the RF signal variation. This reduction can clearly be seen in Supplementary Fig. 9 (red curve).

One way to detect the US envelope is to use an analogue envelope detector. Typically, a circuit implementation of an analogue envelope detector is costly in terms of area and power. According to [2] a simple and effective approximation of the envelope E was found to be

$$\begin{aligned} E\left( t \right)=\max\left( \left| r\left( t \right) \right|, | r_{H}\left( t \right) \right)+\frac{1}{2}\min(\left| r\left( t \right) \right|, |r_{H\left( t \right)}|)\#\left( S2 \right) \end{aligned}$$

where r(t) is the RF ultrasound input, and r_H_(t) is the RF ultrasound input with a 90 degree phase shift relative to the signal centre frequency. Implementing |min(r(t), r_H_(t)| / 2 in the analogue domain is still not very efficient. However it was found in simulations that leaving out this second term has a negligible impact on the image quality. Hence, we estimate the envelope more simply as

$$\begin{aligned} E\left( t \right)=\max(\left| r\left( t \right) \right|, \left| r_{H}\left( t \right) \right|)\#\left( S2 \right) \end{aligned}$$

It is common in US systems to sample data at 4x the fundamental US frequency [10-12]. By doing so, the phase difference between the current and previous sample is 90 degrees and thus Eq. (S2) can be rewritten as

$$\begin{aligned} E\left( t \right)=\max\left( \left| x[n] \right|, | x[n-1]| \right)\#\left( S3 \right) \end{aligned}$$

where n is an integer and indicates the sample index.

The DAC (Supplementary Fig. 8) is used to represent an estimate of the envelope E[n]. When E[n] > DAC[n], U/D goes high and the DAC code is doubled using a bit shift. Likewise, when E[n] < DAC[n], U/D is low and the DAC code is halved. By using the definition of E(t) described by Eq. (S3), the following algorithm can be devised, which uses an intermediate Boolean variable B:

- B[i] = (x[n-i] > 0 AND x[n-i] > DAC[n]) OR (x[n-i] < 0 AND x[n-i] < DAC[n]

- If B[0] or B[1] 🡪 U/D is high, increase DAC code

- Otherwise U/D is low, decrease DAC code

This logic is implemented in ‘Envelope detector’ block shown in Supplementary Fig. 8.

**Supplementary Fig. 9 | Ultrasound signal and its envelope**

**Supplementary Note 7. Dynamic range measurements of the log-delta ADC**

To test the dynamic range of the ADC, it is not possible to measure it using a sine wave due to the logarithmically spaced quantization levels of the ADC. To correct for these logarithmically spaced quantization levels, we apply pre-distortion to the input signal. Similar to what was done in Fig. 4e, where an exponential signal was applied to obtain a linear sweep of the ADC codes, here we use an exponential pre-distortion. The following was found to be able to provide a suitable transformation

$$\begin{aligned} y_{in}=\frac{e^{c_{1}\cdot x}-e^{-c_{1}\cdot x}}{e^{c_{1}}},\#\left( S4 \right) \end{aligned}$$

where x is a sinusoidal signal and c_1_ is a constant which controls the slope of the signal around x = 0. For this constant, 7 was found to be a good value. This results in the waveform shown in Supplementary Fig. 10a. To remove the pre-distortion after conversion, the inverse of S4 is applied to the output of the ADC, which is equal to

$$\begin{aligned} y_{out}=\frac{\ln\left( \frac{1}{2}\cdot\sqrt{\left( y_{adc}^{2}+c_{1}^{2} \right)+y_{adc}\cdot c_{1}} \right)}{c_{1}} \#\left( S5 \right) \end{aligned}$$

The resulting measured Signal to Noise and Distortion Ratio (SNDR) is shown in Supplementary Fig. 10b. Note that the maximum reached SNDR is only 21.9 dB, which is due to the 22 logarithmically spaced levels with which the signal is digitized. This is relatively low compared to other ADCs used in ultrasound systems. However, thanks to the logarithmic compression, the dynamic range is much larger, and is equal to 57 dB (Supplementary Fig. 10b). According to our simulations, the dynamic range value is the most important parameter of the ADC for achieving images with high SSIM compared to the original.

57 dB

**Supplementary Fig. 10 | ADC characterization. a**, pre-distorted sine wave used for calculating the SNDR of the ADC. **b**, measured and simulated SNDR for different input amplitudes.

**Supplementary Note 8. Additional validation of the proposed log-delta ADC**


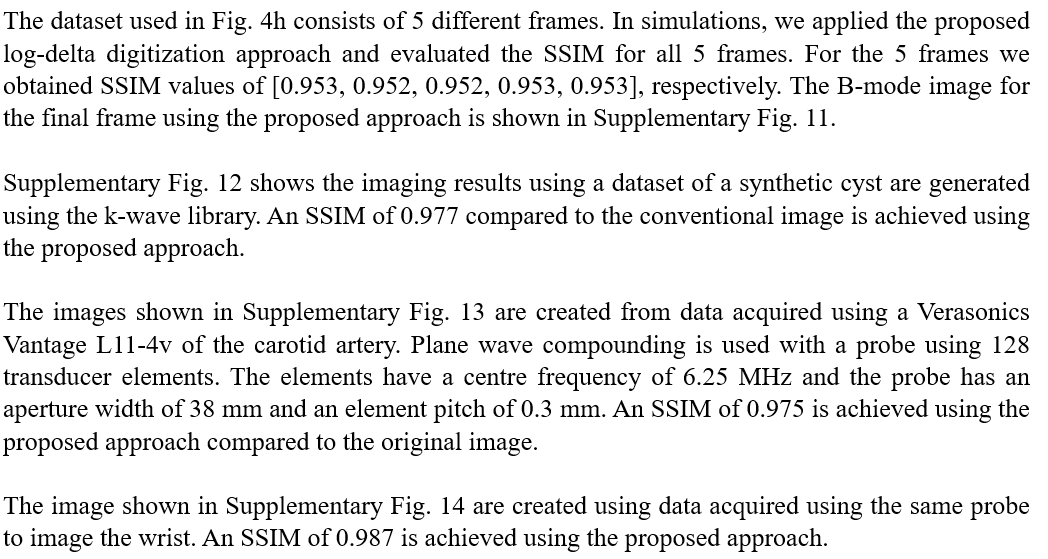


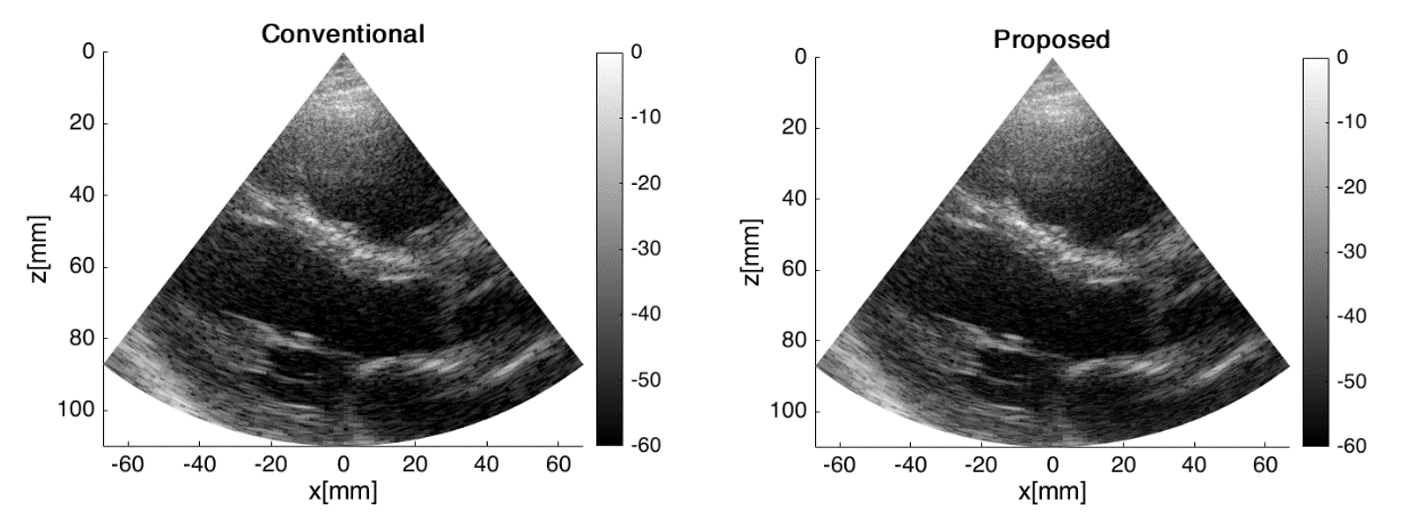


**Supplementary Fig. 11 Simulated B-mode image of the human heart**

**Supplementary Fig. 12 Simulated B-mode image of a synthetic cysts**

**Supplementary Fig. 13 Simulated B-mode image of the carotid artery**

**Supplementary Fig. 14 Simulated B-mode image of the wrist**

**

**Supplementary Fig. 15 B-mode image captured using plane-wave imaging after performing 10 averages, and scaling the image with a 50 dB dynamic range from the brightest point. Hyperbolic artifacts around the copper wires can be clearly observed as a result of the single- angle plane wave transmission. These artifacts are expected to diminish significantly when coherent plane wave compounding or other focused transmit beamforming techniques are employed.**

**Supplementary Note 9. US TX power consumption**

In this work, a commercially available ultrasound pulser (see Methods section) was used, which is capable of sourcing and sinking 2.5 A. This is well beyond the requirements of our system, and contributes to the rather high TX power consumption measured in our system, which is reported in Fig. 5d.

To quantify in a fair way the US TX energy needs for this system, the energy required to drive the TX transducers can be calculated using the equation E = CV^2^. For this prototype, the total capacitance (C) driven by the pulser is 15 pF and the driving voltage is 200 V_pp_, resulting in 600 nJ per pulse. Dividing this by 64 (the number of TX transducers) gives approximately 9.4 nJ per transducer per shot.

Due to the 8-1 multiplexing used in this work, the TX pulse needs to be generated 8 times. Therefore, the energy associated with the TX is also 8 times larger, compared to a system not using multiplexing. To put this energy requirement in comparison in perspective: keeping the receive frontend active for 250 µs (the acquisition time needed after a single TX shot) results in about 337 nJ of energy consumption. The 8 shots for the US transmission consume 75.2 nJ.

Supplementary Table 1 shows all contributions to the power consumption in our system, comparing the power that would be required from a conventional implementation with the one actually measured in the proposed system.

**Supplementary Table 1**. Power consumption breakdown per channel for a conventional implementation and using the power reduction techniques proposed in our system

|  | **Conventional (mW)** | **Proposed (mW)** |
| --- | --- | --- |
| US TX | 3.38 | 3.38 |
| Data TX | 18.0 | 3.60 |
| ADC | 0.30 | 0.14 |
| Rest of AFE | 0.56 | 0.56 |
| TIA | 1.50 | 0.66 |

**Supplementary Note 10. Benchmarking**

The proposed ASIC is compared to other ultrasound ASICs targeting similar transducers. The design is benchmarked against academic literature [3-5] and a commercial component [6]. This table compares the active area and power consumption of the ADC of our solution to other designs that do not implement compression, highlighting that our architecture does not add significant power compared to systems not implementing compression. To facilitate comparison, we have also included the simulated voltage noise density of A1 (Fig. 3a, opamp used in the TIA). This allows comparison of the noise performance of our system to systems implementing an LNA instead of a TIA.

|  | This work | [3] | [4] | [5] | [6] |
| --- | --- | --- | --- | --- | --- |
| Technology | 65 nm LV CMOS | 180 nm BCD | 180 nm BCD | 40 nm LV CMOS | N.A. |
| Transducer | 1D PVDF | 2D PZT | 2D PZT | 1D CMUT | N.A. |
| Array size | 1 x 32 | 16 x 18 | 8 x 9 | 1 x 96 | 1 x 32 |
| Element capacitance ($C_{el})$ | 10 pF^a^ | 1 pF | N.A. | 25 pF | N.A. |
| Element pitch | 180 µm | 160 µm | 160 µm | 102 µm | N.A. |
| Max TX voltage | 200 V | 30 V | 65 V | 50 V | N.A. |
| RX architecture | TFT MUX + AFE + ADC + Datalink | AFE + µBF + ADC + Datalink | AFE + µBF + ADC + Datalink | On-chip MUX + AFE + ADC + Datalink | AFE + ADC |
| Active area of ADC | 0.01 mm^2^ | 0.007 mm^2^ | 0.01 mm^2^ | N.A. | N.A. |
| Active area of AFE+ADC | 0.04 mm^2^ | 0.03 mm^2^ | 0.032 mm^2^ | N.A. | N.A. |
| Peak SNR | 49.8 dB | 52.2 dB | 52.3 dB | 50.4 dB | 72 dB |
| Sample rate | 32 MHz | N.A. | 24 MHz | 80 MHz | 40 MHz |
| AFE bandwidth ($f_{BW})$ | 12 MHz | N.A. | N.A. | 18 MHz | 10 MHz – 18 MHz  (programmable) |
| Average AFE power consumption ($P_{AFE})$ | 1.21 mW^b^ | 0.3 mW | 0.55 mW | 2.49 mW | N.A. |
| ADC power consumption | 0.14 mW | 0.36 mW | 0.51 mW | 0.18 mW | N.A. |
| AFE + ADC average power consumption | 1.35 mW | 0.66 mW | 1.06 mW | 2.67 mW | 0.76 mW |
| Input referred noise of AFE ($i_{n,rms})$ | 12.9 nA_rms_ ^d^  5.5 nA_rms_ ^e^ | N.A. | N.A. | 12.5 nA_rms_ | N.A. |
| Input referred voltage noise density of AFE ^c^ | 5 nV/√Hz ^d^ 2.5 nV/√Hz ^e^ | N.A. | 12.7 nV/√Hz | N.A. | 4 nV /√Hz |
| AFE power consumption in the two modes | 1.01 mW ^d^  2.06 mW ^e^ | 0.3 mW | 0.55 mW | 2.49 mW | N.A. |

^a^ Estimated parasitic capacitance of interconnection on PCB

^b^ The average power is calculated summing 4/5^th^ of the high noise consumption and 1/5^th^ of low noise mode consumption

^c^ To facilitate comparison, we have also included the simulated voltage noise density of A_1_ (Fig. 3a, opamp used in the TIA)

^d^ High noise mode

^e^ Low noise mode

**Supplementary references**

[1] M. van Elzakker, E. van Tuijl, P. Geraedts, D. Schinkel, E. Klumperink and B. Nauta, "A 1.9μW 4.4fJ/Conversion-step 10b 1MS/s Charge-Redistribution ADC," 2008 IEEE International Solid-State Circuits Conference - Digest of Technical Papers, San Francisco, CA, USA, 2008, pp. 244-610

[2] M. Schlaikjer, J. P. Bagge, O. M. Sorensen and J. A. Jensen, "Trade off study on different envelope detectors for B-mode imaging," IEEE Symposium on Ultrasonics, 2003, Honolulu, HI, USA, 2003, pp. 1938-1941 Vol.2

[3] Y. M. Hopf, D. S. dos Santos, B. W. Ossenkoppele, M. Soozande, E. Noothout, Z.-Y. Chang,

C. Chen, H. J. Vos, J. G. Bosch, M. D. Verweij, N. de Jong, and M. A. P. Pertijs, “A Pitch-

Matched High-Frame-Rate Ultrasound Imaging ASIC for Catheter-Based 3-D Probes,” IEEE

Journal of Solid-State Circuits, vol. 59, no. 2, pp. 476–491, 2024.

[4] Y. M. Hopf, B. W. Ossenkoppele, M. Soozande, E. Noothout, Z.-Y. Chang, C. Chen, H. J. Vos,

J. G. Bosch, M. D. Verweij, N. de Jong, and M. A. P. Pertijs, “A Pitch-Matched Transceiver

ASIC With Shared Hybrid Beamforming ADC for High-Frame-Rate 3-D Intracardiac Echocar-

diography,” IEEE Journal of Solid-State Circuits, vol. 57, no. 11, pp. 3228–3242, 2022.

[5] M. Zhou, P. Chen, A. M. A. O. Pollet, S. Ouzounov, J. M. J. den Toonder, M. Mischi, E. Can-

tatore, and P. Harpe, “A Prototype System With Custom-Designed RX ICs for Contrast-

Enhanced Ultrasound Imaging,” IEEE Transactions on Ultrasonics, Ferroelectrics, and Fre-

quency Control, vol. 69, no. 5, pp. 1649–1660, 2022

[6] Texas Instruments, AFE5832LP: 32-Channel Ultrasound Analog Front End with Digital Demodulator, Datasheet, Rev. A, Dec. 2020. [Online]. Available: <https://www.ti.com/product/AFE5832LP>

[7] K. Ishida et al., "Radio frequency electronics in a-IGZO TFT technology," 2016 23rd International Workshop on Active-Matrix Flatpanel Displays and Devices (AM-FPD), Kyoto, Japan, 2016, pp. 273-276

[8] P. Guo et al., "A Pitch-Matched Low-Noise Analog Front-End With Accurate Continuous Time-Gain Compensation for High-Density Ultrasound Transducer Arrays," in IEEE Journal of Solid-State Circuits, vol. 58, no. 6, pp. 1693-1705

[9] G. Szentirmai, “Synthesis of multiple-feedback active filters,” The Bell System Technical Journal , vol. 52, no. 4, pp. 527-555, 1973.

[10] Texas Instruments, “Signal Processing Overview of Ultrasound Systems for Medical Imaging,” November 2008. [Online]. Available: https://www.ti.com/lit/wp/sprab12/sprab12.pdf. [Accessed 5 12 2024].

[11] C. Madiena, J. Faurie, J. Porée and D. Garcia, “Color and Vector Flow Imaging in Parallel Ultrasound With Sub-Nyquist Sampling,” IEEE Transactions on Ultrasonics, Ferroelectrics, and Frequency Control, vol. 65, no. 5, pp. 795-803, 2018.

[12] N. Wagner, Y. C. Eldar, A. Feuer, G. Danin and Z. Friedman, “Xampling in Ultrasound Imaging,” in SPIE Medical Imaging, Orlando, 2011.
